# Prevalence of Acute Pediatric Burns in a Tertiary Care Hospital

**DOI:** 10.31729/jnma.5233

**Published:** 2020-11-30

**Authors:** Kiran Kishor Nakarmi, Bishnu Deep Pathak

**Affiliations:** 1 Department of Burns, Plastic and Reconstructive Surgery, Kirtipur Hospital, Kathmandu, Nepal; 2 Nepalese Army Institute of Health Sciences, Bhandarkhal, Kathmandu, Nepal

**Keywords:** *burns*, *pediatric*, *prevalence*

## Abstract

**Introduction:**

Burn injury is an important cause of mortality and morbidity in children worldwide. Mortality is higher in developing countries than in developed ones. Most of them occur in predictable domestic settings and can be prevented. The objective of this study was to find out the prevalence of acute pediatric burns in a hospital setting.

**Methods:**

A descriptive cross-sectional study was conducted by reviewing the secondary data of burn cases admitted during the years 2016 AD to 2018 AD in a tertiary care hospital after taking ethical clearance from the Institutional Review Committee (IRC No. 016-2019). The sample size was calculated and systematic random sampling was done. Data analysis was done using Statistical Package for the Social Sciences, version 23. Point estimate at 95% Confidence Interval was calculated along with frequency and proportion for binary data.

**Results:**

The prevalence of acute pediatric burns at the hospital was found to be 101 (29.71%) (24.85-34.57 at 95% Confidence Interval). The majority of them were males 54 (53.47%) and toddlers 39 (38.61%). Scalding 54 (53.47%) was the main etiology. Most of the burn injuries occurred inside the house 76 (75.25%) and on November 11 (10.9%). The mortality rate was 11 (10.89%).

**Conclusions:**

The prevalence of acute pediatric burns in a hospital setting was lower than most other countries but mortality was higher. The majority of the burn injuries occurred inside the house. Therefore, special focus should be done on prevention at the household level. Adequate medical services for emergency management of childhood burns should be available in different parts of the country.

## INTRODUCTION

Pediatrics are individuals less than 18 years of age.^1^ Burn injury in the pediatric population is a global health problem.^2^ It is an important cause of significant mortality and morbidity in this age group.^3^ Children have unique biological responses to burn injuries. This demands a different approach to resuscitation and management.^4^ The risk of mortality in children from burns is higher than that in adults.^5^

The pediatric burn is a huge challenge for developing countries where there is a higher risk of burns and treatment difficulties.^3^ Mortality associated with burn injuries is higher in developing countries than developed ones.^5^ Childhood burns mostly occur in predictable domestic settings and most of them can be prevented.^3,6^ So, it is important to conduct programs at the national level for the prevention and management of burn injuries specific tothe pediatric population.

The main objective of this study was to find out the prevalence of acute pediatric burns among admitted burn cases at Kirtipur Hospital, Kathmandu over three years (2016 to 2018 AD).

## METHODS

A descriptive cross-sectional study was conducted by reviewing the secondary data of burn cases admitted during the years 2016 AD to 2018 AD in Kirtipur Hospital, Kathmandu. Ethical approval was taken from the Institutional Review Committee (IRC No. 0162019), phect-NEPAL. Secondary data were collected from hospital records which included all the acute burn cases admitted in the Plastic Surgery ward of Kirtipur Hospital, Kathmandu from the years 2016 to 2018 AD. Admitted burn cases with inadequate information and burn cases managed in the emergency room only and as an outpatient basis was excluded from the study. Systematic random sampling was done.

The sample size was calculated as follows;

$$\begin{array}{ll} \text{Sample size (n)} & \text{= }{\text{Z}}^{\text{2}}\times \text{p}\times \text{(1}-\text{p)}/{\text{e}}^{\text{2}}\\ & \text{= }{\left(1.96\right)}^{\text{2}}\times (0.5\times 0.5)/\text{0}{\text{.06}}^{\text{2}}\\ & \text{= }264 \end{array}$$

where,

n = required sample size
Z = 1.96 at 95% Confidence Interval (CI)
p = prevalence taken, 50%
e = margin of error, 6%

The minimum required sample size was 264. But 304 patient cases were taken into the study.

Data analysis was done in Statistical Package for the Social Sciences, version-23. Period prevalence at a 95% confidence interval was calculated.

## RESULTS

The prevalence of acute pediatric burns at Kirtipur Hospital, Kathmandu from 2016 AD to 2018 AD was found to be 101 (29.71%) (24.85-34.57 at 95% CI).

Among total pediatric burn cases, 54 (53.47%) were males and 47 (46.53%) were females. The median age of children was 4 years. Most of them were toddlers 39 (38.61%) followed by school-age child 22 (21.78%) (Table 1).

**Table 1:** Age-wise distribution of pediatric burns.

Age group	Frequency n (%)
Less than 12 months (Infants)	9 (8.91)
12 months to 36 months (Toddlers)	39 (38.61)
37 months to 72 months (Pre-school child)	20 (19.80)
73 months to 12 years (School-age child)	22 (21.78)
More than 12 years	11 (10.89)

**Figure 1:**
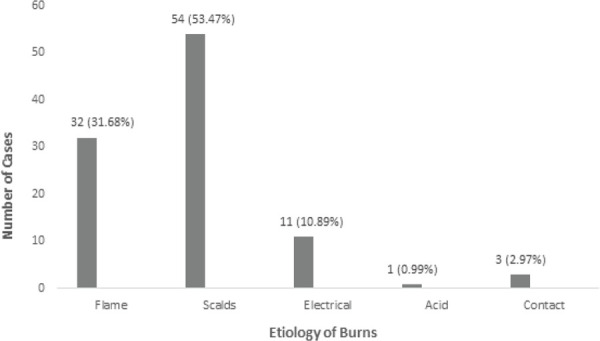
Etiology of pediatric burns.

The main etiology of burns was scalding 54 (53.47%) followed by flame burns 32 (31.68%) (Figure 1).

Maximum burn incidents took place in the month of November 11 (10.9%) followed by January 10 (9.9%) and March 10 (9.9%) (Table 2).

**Table 2:** Month-wise distribution of pediatric burns.

Months	n (%)
January	10 (9.9)
February	8 (7.9)
March	10 (9.9)
April	9 (8.9)
May	8 (7.9)
June	5 (5.0)
July	7 (6.9)
August	8 (7.9)
September	8 (7.9)
October	9 (8.9)
November	11 (10.9)
December	8 (7.9)

The majority 76 (75.25%) burn injuries occurred inside the house. Only 25 (24.75%) incidents took place outside the house.

The median Total Body Surface Area (TBSA) of burns was 10% with a maximum being 90% and the minimum being 0.05%. The mean duration of hospital stay was 11.15 days with the maximum and minimum duration being 96 days and 0 days respectively.

Eleven out of 101 cases died with a mortality rate being 10.89%.

## DISCUSSION

There are several studies on pediatric burns all around the world. According to our study, the prevalence of acute pediatric burns in a hospital setting is 29.71%. This is comparable to the prevalence (28.6%) in a burn unit in Jinzhou, China.^7^ Another study in Nepal^8^ showed the prevalence of burns in the 0-15 years age group to be 25.6% which is similar to ours. Similarly, our finding corresponds to the finding in Nigeria where the proportion of childhood burns is 33.3%.^9^

But this is contrary to the findings in Western Regional Hospital of Nepal where the prevalence in 0-15 years children is 61%.^10^ Similarly, studies in hospitals of Europe show that burns in children account for half of all burn injuries.^11^ Pediatric burns alone cover 40% of all admissions every year at three burn centers in Netherland.^12^ Approximately, 50% of the burn cases in the United States are younger than five years of age.^13^

In our study, mortality due to pediatric burns is 10.89% which is higher than that in Shanghai, China,^14^ and Saudi Arabia.^15^ Majority of the pediatric cases in our study are males and the burn injury occurred inside the house which is similar in South Central China.^16^ The major cause of pediatric burns in our study is scalds followed by flame burns. This is in line with the findings in Israel^2^ and the United States of America (USA).^13^

Our study depicts that pediatric burns are maximal among toddlers (38.61%). This is supported by studies in South Central China.^16^ But, in Iran^6^ and Israel,^2^ it is maximum among preschool-age children and infants respectively. In Hong Kong, out of total hospital admissions for burns, 42.7% belonged to toddlers of age less than two years.^17^ In the same way, in Israel, 51% of all burn admissions comprised of children (0-14 years) with infants (0-1 year) having the highest prevalence (45%).^2^

There are some limitations to our study that need to be mentioned. First, it is conducted in a hospital setting only which cannot be generalized for the whole country. Next, secondary data of only three year period is taken and the burn cases managed in the emergency room and as an out-patient basis were excluded.

## CONCLUSIONS

The prevalence of acute pediatric burns in a hospital setting was found to be lower than most other countries. The majority of the cases were male toddlers with scalds being the major etiology. Most of the burn injuries occurred inside the house in the month of November. Mortality was higher than in other countries. So, special focus should be given on the prevention of pediatric burns at the household level. Besides it, there should be the provision of adequate medical services for emergency management of childhood burn injuries in different parts of the country.

## References

[1] Paul VK, Bagga A (2018). Ghai Essential Pediatrics.

[2] Goldman S, Aharonson-Daniel L, Peleg K (2006). Childhood burns in Israel: a 7-year epidemiological review. Burns.

[3] Delgado J, Ramirez-Cardich ME, Gilman RH, Lavarello R, Dahodwala N, Bazan A (2002). Risk factors for burns in children: crowding, poverty, and poor maternal education. Inj Prev.

[4] Arbuthnot MK, Garcia AV (2019). Early resuscitation and management of severe pediatric burns. Semin Pediatr Surg.

[5] Agbenorku P, Agbenorku M, Fiifi-Yankson PK (2013). Pediatric burns mortality risk factors in a developing country's tertiary burns intensive care unit. Int J Burns Trauma.

[6] Rafii MH, Saberi HR, Hosseinpour M, Fakharian E, Mohammadzadeh M (2012). Epidemiology of pediatric burn injuries in isfahan, iran. Arch Trauma Res.

[7] Hai Jun W, Jie X, Jun Z, Feng T, Hui HG (2011). Comparable results of epidemiology of children with burns among different decades in a burn unit in JinZhou, China. Burns.

[8] Rai SM, Karki B, Nakarmi K, Ghartimagar M, Nagarkoti K, Joshi KD (2014). Retrospective study on early outcome of acute burn injuries treated at Nepal Cleft and Burn Centre of Public Health Concern Trust-Nepal. J Nepal Health Res Counc.

[9] Dongo AE, Irekpita EE, Oseghale LO, Ogbebor CE, Iyamu CE, Onuminya JE (2007). A five-year review of burn injuries in Irrua. BMC Health Serv Res.

[10] Liu EH, Khatri B, Shakya YM, Richard BM (1998). A 3 year prospective audit of burns patients treated at the Western Regional Hospital of Nepal. Burns.

[11] Kemp AM, Jones S, Lawson Z, Maguire SA (2014). Patterns of burns and scalds in children. Arch Dis Child.

[12] Baartmans MG, De Jong AE, Van Baar ME, Beerthuizen GI, Van Loey NE, Tibboel D (2016). Early management in children with burns: Cooling, wound care and pain management. Burns.

[13] Kliegman RM, Stanten BF, Schor NF, Geme JW (2016). Nelson Textbook of Pediatrics.

[14] Xin W, Yin Z, Qin Z, Jian L, Tanuseputro P, Gomez M (2006). Characteristics of 1494 pediatric burn patients in Shanghai. Burns.

[15] Jamal YS, Ardawi MS, Ashy AR, Shaik SA (1990). Paediatric burn injuries in the Jeddah area of Saudi Arabia: a study of 197 patients. Burns.

[16] Zhou B, Zhou X, Ouyang LZ, Huang XY, Zhang PH, Zhang MH (2014). An epidemiological analysis of paediatric burns in urban and rural areas in south central China. Burns.

[17] Ying SY, Ho WS (2001). An analysis of 550 hospitalized pediatric burn patients in Hong Kong. J Burn Care Rehabil.

